# The Effects of T4 and A3/R Bacteriophages on Differentiation of Human Myeloid Dendritic Cells

**DOI:** 10.3389/fmicb.2016.01267

**Published:** 2016-08-17

**Authors:** Katarzyna Bocian, Jan Borysowski, Michał Zarzycki, Magdalena Pacek, Beata Weber-Dąbrowska, Maja Machcińska, Grażyna Korczak-Kowalska, Andrzej Górski

**Affiliations:** ^1^Department of Immunology, Faculty of Biology, University of WarsawWarsaw, Poland; ^2^Department of Clinical Immunology, Transplantation Institute, Medical University of WarsawWarsaw, Poland; ^3^Laboratory of Bacteriophages, Ludwik Hirszfeld Institute of Immunology and Experimental Therapy, Polish Academy of SciencesWrocław, Poland

**Keywords:** bacteriophage, T4, A3/R, phage therapy, dendritic cell, differentiation, lysate

## Abstract

Bacteriophages (phages) are viruses of bacteria. Here we evaluated the effects of T4 and A3/R bacteriophages, as well as phage-generated bacterial lysates, on differentiation of human myeloid dendritic cells (DCs) from monocytes. Neither of the phages significantly reduced the expression of markers associated with differentiation of DCs and their role in the activation of T cells (CD40, CD80, CD83, CD86, CD1c, CD11c, MHC II, PD-L1, PD-L2, TLR2, TLR4, and CCR7) and phagocytosis receptors (CD64 and DEC-205). By contrast, bacterial lysate of T4 phage significantly decreased the percentages of DEC-205- and CD1c-positive cells. The percentage of DEC-205-positive cells was also significantly reduced in DCs differentiated in the presence of lysate of A3/R phage. Thus while bacteriophages do not substantially affect differentiation of DCs, some products of phage-induced lysis of bacterial cells may influence the differentiation and potentially also some functions of DCs. Our results have important implications for phage therapy of bacterial infections because during infections monocytes recruited to the site of inflammation are an important source of inflammatory DCs.

## Introduction

Bacteriophages (phages) are viruses of bacteria. Phages constitute an extremely abundant and diversified group of viruses. Thus far, over 6000 bacteriophages have been identified by using electron microscopy and the total number of phage virions in the biosphere has been estimated at around 10^32^. Phages occur in great numbers in different environments including water, soil, and air. Moreover they are an important component of the microflora in humans and different animals (Letarov and Kulikov, 2009; Hatfull and Hendrix, 2011; Ackermann and Prangishvili, 2012; Dalmasso et al., 2014). The main biomedical application of bacteriophages is the treatment of bacterial infections; many studies performed on animal models of infections, observational clinical studies, and the first small randomized controlled trials of phage therapy indicate high safety and efficacy of phages as antibacterial agents (Kutter et al., 2014; Wittebole et al., 2014). In addition, a growing body of data shows that bacteriophages can affect functions of different populations of immune cells involved in both innate and adaptive immunity including neutrophils, monocytes, macrophages, as well as T and B cells (Borysowski et al., 2010; Górski et al., 2012).

An important population of immune cells critically involved in the regulation of immune responses are dendritic cells (DCs). DCs present antigens to lymphocytes, priming naïve T cells and driving them toward a specific lineage fate. The maturation state of DCs can be manipulated to boost or suppress immune responses. Several DC subpopulations have been identified based on typical surface molecule expression patterns. Each DC subset possesses unique functional specialization. The major population of human myeloid DCs (mDCs) in blood, tissues and lymphoid organs are CD1c^+^ mDCs. They were originally identified in blood as a fraction of HLA-DR^+^ lineage cells expressing myeloid antigens CD11b, CD11c, CD13, CD33, CD172a (SIRP α) and CD45RO. Human tissue CD1c^+^ DCs tend to be more activated than their blood counterparts in terms of expression of CD80, CD83, CD86 and CD40. They have lost expression of homing receptors CLA and CD62L but up-regulated CCR7. CD1c^+^ DCs are equipped with a wide range of lectins, Toll-like receptors (TLRs) and other pattern recognition receptors (PRRs) involved in antigen uptake, transport and presentation (Collin et al., 2013; McGovern et al., 2015; Poltorak and Schraml, 2015**;** Vu Manh et al., 2015).

Given the central role of DCs in the regulation of immune responses, research on the interactions between phages and DCs is essential for understanding the effects of bacterial viruses on the immune system. Over recent years the first studies have been conducted to investigate the influence of bacteriophages on some functions of DCs (Pajtasz-Piasecka et al., 2008**;** Sartorius et al., 2011**;** Miernikiewicz et al., 2013**;** An et al., 2014). All these studies were performed on murine bone marrow-derived dendritic cells (BMDCs); to the best of our knowledge, there are no data on the interactions between phages and human DCs. The results of studies of murine DCs cannot be simply extrapolated to human DCs because of substantial differences between functions of murine and human DCs; these differences pertain to the production of some cytokines, the expression of some surface molecules including TLRs, and the effects of viral stimulation (Robbins et al., 2008**;** Schlitzer et al., 2015). Therefore, in this study we asked whether bacteriophages can affect human DCs.

The main objective of this study was to evaluate the effects of two bacteriophages – T4 (specific for *Escherichia coli*) and A3/R (specific for *Staphylococcus aureus*) on the differentiation of human mDCs from monocytes *in vitro*. Moreover, we investigated whether mDCs differentiation can be affected by the products of phage-induced lysis of host bacteria.

## Materials and Methods

### Bacteriophages

T4 phage was purchased from American Type Culture Collection (ATCC; USA) and was cultured on *E. coli* B obtained from the Collection of Microorganisms of the L. Hirszfeld Institute of Immunology and Experimental Therapy (IIET), Wrocław, Poland.

A3/R was obtained from the Phage Collection of IIET. It is a descendant of A3 phage which was originally obtained from Gerhard Pulverer, Institute of Hygiene, University of Cologne, Germany in 1986, and adapted to therapeutic use by several rounds of passaging through clinical *S. aureus* strains. A3/R was cultured on *S. aureus* strain 19930 from Laboratory of Bacteriophages, IIET.

Purified preparation of T4 phage was prepared by Laboratory of Bacteriophages, IIET, as described in detail elsewhere (Boratyński et al., 2004). In brief, the protocol involves sequential ultrafiltration of crude phage-generated *E. coli* lysate through polysulfone membranes followed by chromatography on sepharose 4B (Sigma-Aldrich, Poland) and cellulofine sulfate (Millipore, USA). To obtain purified preparation of A/3R, crude phage-generated *S. aureus* lysate was subjected to ultrafiltration through polysulfone membranes and chromatography on sepharose 4B. Stock phage preparations were suspended in phosphate-buffered saline (PBS). Phage titer was measured by two-layer method of Adams (1959). The concentration of LPS in purified phage preparations was determined using QLC-1000 Endpoint Chromogenic LAL test kit (Lonza) according to the manufacturer’s instructions. In the A3/R preparation the concentration of LPS was undetectable, while in the T4 phage preparation it was 3 ng/ml. Therefore, as an additional control for T4 phage, LPS (Sigma-Aldrich, Poland) was used at a concentration of 3 ng/ml. LPS was diluted with PBS.

### Bacterial Lysates

Phage-generated bacterial lysates were prepared by Laboratory of Bacteriophages, IIET, according to the modified method by Slopek et al. (1983; Letkiewicz et al., 2009). In brief, phages were incubated with their host bacteria in LB medium at 37°C until complete bacterial lysis occurred (approx. 4–6 h). Subsequently the suspension was filtered through a 0.22-μm filter (Millipore, USA). Stock preparations of both lysates were suspended in peptone water (IIET, Poland). Phage titer in lysates was measured by two-layer method of Adams (1959).

As an additional control in experiments to evaluate the effects of lysates on differentiation of DCs peptone water was used.

### Generation of Myeloid Dendritic Cells from Peripheral Blood Monocytes

The study was performed on cells isolated from healthy blood donors. Informed, written consent was obtained from all donors and the study was approved by the ethical committee of the Medical University of Warsaw. Peripheral blood mononuclear cells (PBMCs) were isolated from blood specimens by density-gradient centrifugation over Gradisol L (Aqua Medica, Łódź, Poland). Isolation of monocytes from PBMCs was performed using Dynabeads^®^ FlowComp^TM^ Human CD14 (Invitrogen) according to the manufacturer’s instructions. The purity of monocyte population was evaluated by flow cytometry (FACSCalibur, Becton Dickinson) using anti-CD14 monoclonal antibody (BD Pharmingen). The purity of the isolated monocytes was 98%. Monocytes were cultured at a density of 1 × 10^6^/ml in the AIM-V medium (Gibco) supplemented with IL-4 (100 ng/ml; R&D Systems) and GM-CSF (250 ng/ml; R&D Systems) in 24-well plates at 37°C with 5% CO_2_ for 5 days. In some cultures purified phage preparations or bacterial lysates were added on day 0 to wells at a density of 10^8^ PFU/ml (final concentration). In control cultures, equal volumes of PBS were added to wells. Differentiation of monocytes into mDCs was evaluated by flow cytometry (FACSCalibur, Becton Dickinson) using the following monoclonal antibodies: anti-CD11c-APC (BD Pharmingen), anti-CD1c-FITC (eBioscience), anti-CCR7-APC-e Fluor (eBioscience). In addition, cells were stained with anti-CD14-PerCP (BD Pharmingen) to confirm the state of differentiation of DCs which lose CD14 during differentiation. We consistently found that the percentage of CD14 cells in cultures was 98%. In addition, cell morphology during DCs differentiation was analyzed using ECLIPSE TE200 inverted microscope (Nikon) with a digital camera and the NIS-Elements software. On the last day of culture viability of DCs was determined using trypan blue. The viability of cells was 95%. Cultured cells were photographed and the expression of the investigated markers was determined by flow cytometry.

### Evaluation of Expression of mDCs Surface Markers

Cells were incubated with appropriate monoclonal anti bodies: CD11c-APCw (BD Pharmingen), CD1c-FITC (eBioscience), CD83-PE (BD Pharmingen), CD80-FITC (BD Pharmingen), CD86-PE (BD Pharmingen), CD40-PE (BD Pharmingen), PD-L1-FITC (BD Pharmingen), PD-L2-PE (BD Pharmingen), HLA-DR-PE (BD Pharmingen), CCR7-APC-e Fluor (eBioscience), TLR2-FITC (eBioscience), TLR4-PE (eBioscience), CD64-FITC (BD Pharmingen), and DEC-205-PE (BD Pharmingen). In addition, CD14-PerCP (BD Pharmingen) monoclonal antibody was used to confirm the differentiation of DCs. Isotype controls were cells stained with IgG1 conjugated with the respective fluorochromes (BD Pharmingen, eBioscience). After 30 min of incubation at 4°C, cells were washed twice in FACS buffer (Becton Dickinson). The expression of individual markers on gated CD1c^+^ DCs was determined by flow cytometry (FACSCalibur, Becton Dickinson) and analyzed by Cell Quest software (Becton Dickinson). The results were based on analysis of at least 100,000 cells and were shown as the percentage of positively labeled cells. In addition, the mean channel fluorescence values of gated DCs positive for individual markers were determined.

### Determination of Cytokine Production

The concentration of IL-12 (p70) was measured in cell culture supernatants by enzyme-linked immunoassay (ELISA) using Human IL-12 p70 ELISA Ready-SET-Go kit according to the manufacturer’s instructions (eBioscience).

### Statistics

Statistical analysis was performed by Wilcoxon’s matched pairs test. *P* < 0.05 was regarded as significant.

## Results

To evaluate the effects of T4 and A3/R phages on the differentiation of mDCs, we analyzed the expression of main DC markers on mDCs differentiated from monocytes in the presence of individual preparations. We focused on two classes of markers: (i) molecules associated with maturation of DCs and their role in the activation of T cells (CD40, CD80, CD83, CD86, CD1c, CD11c, MHC class II, PD-L1, PD-L2, TLR2, TLR4, and CCR7), and (ii) receptors involved in phagocytosis (CD64 and DEC-205). In each case, we determined both the percentage of mDCs expressing a given marker and the value of the mean channel fluorescence of gated mDCs positive for this marker.

**FIGURE 1:**
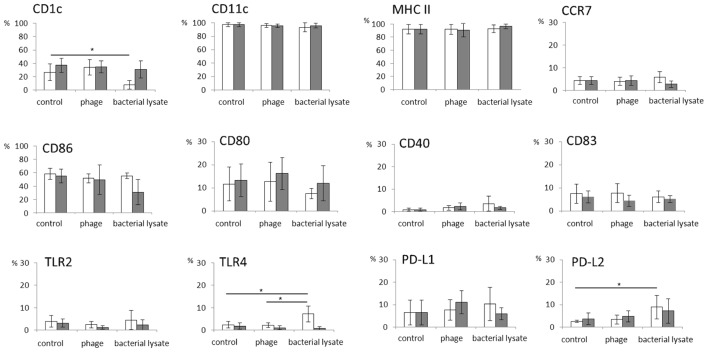
**The effects of bacteriophages and bacteriophage-generated bacterial lysates on the expression of markers associated with the differentiation of myeloid dendritic cells and their role in T cell activation.** Human myeloid dendritic cells (mDCs) were differentiated from monocytes during five-day cultures with IL-4 (100 ng/ml) and GM-CSF (250 ng/ml) in the presence of individual phage preparations including purified preparation of T4 (white bars, 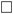), T4-generated *Escherichia coli* lysate (white bars, 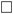), purified preparation of A3/R (gray bars, 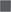), or A3/R-generated *Staphylococcus aureus* lysate (gray bars, 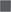). In control cultures cells were treated with equal volumes of PBS. The expression of all markers including CD40, CD80, CD83, CD86, CD1c, CD11c, MHC class II, PD-L1, PD-L2, TLR2, TLR4, and CCR7 was determined by flow cytometry. The bars show the percentages of mDCs expressing individual markers. The experiment was repeated five times. *p < 0.05.

In general, both T4 and A3/R phage had no effect on the expression of any of the investigated markers. The percentages of cells expressing individual markers associated with differentiation of DCs and their role in the activation of T cells were comparable in mDCs generated in the presence of bacteriophages and in control cultures (**Figure 1**). Of note, we found some interpersonal variability in the level of CD80 expression compared with the level of expression of other analyzed markers.

**FIGURE 2:**
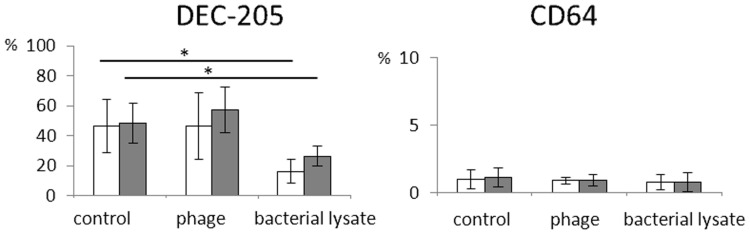
**The influence of bacteriophages and bacteriophage-generated bacterial lysates on the expression on myeloid dendritic cells of receptors involved in phagocytosis.** Human mDCs were differentiated from monocytes during five-day cultures with IL-4 (100 ng/ml) and GM-CSF (250 ng/ml) in the presence of individual phage preparations including purified preparation of T4 (white bars, 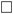), T4-generated *Escherichia coli* lysate (white bars, 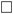), purified preparation of A3/R (gray bars, 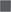), or A3/R-generated *Staphylococcus aureus* lysate (gray bars, 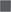). In control cultures cells were treated with equal volumes of PBS. The expression on mDCs of DEC-205 and CD64 molecules was determined by flow cytometry. The bars show the percentages of mDCs expressing DEC-205 and CD64. The experiment was repeated five times. ^∗^*p < 0.05.*

Likewise, bacteriophages had no effect on the percentages of CD64- and DEC-205-positive mDCs (**Figure 2**). In addition, we found no significant differences between the expression of the analyzed markers on cells cultured in the presence of LPS (an additional control for T4 phage) and cells from control cultures (treated with PBS; data not shown).

In addition, we found no significant differences in the mean channel fluorescence values for the analyzed markers between mDCs differentiated in the presence of phages and in control cultures (**Table 1**).

**Table 1:** Geo mean values of surface markers of mDCs differentiated in the presence of bacteriophages and bacteriophage-generated bacterial lysates.

	Control		phage		bacterial lysate	
Geo Mean	T4	A3/R	T4	A3/R	*Escherichia coli* (T4)	*Staphylococcus aureus* (A3/R)
CD1c	23.2 (± 4.3)	24.7 (± 9.5)	23.6 (± 4.2)	24.8 (± 7.7)	20.6 (± 3.5)	22.8 (± 4.7)
CD11c	182.5 (± 67.3)	134.4 (± 36.3)	140.5 (± 38.5)	119.7 (± 32.4)	111.3 (± 23.3)	137.6 (± 35.4)
MHCII	77.2 (± 34)	50.2 (± 17.5)	70 (± 9.5)	47.6 (18.5)	60.2 (± 14.9)	47.3 (± 8.1)
CCR7	36.3 (± 3.2)	46.4 (± 22.8)	35.8 (± 14.1)	58.8 (± 30.7)	50.3 (± 31.7)	43.8 (± 20.3)
CD86	62.5 (± 14.1)	63.8 (± 12.5)	65 (± 10.4)	67 (± 13.4)	57.8 (± 4.3)	57 (± 12.9)
CD80	51.6 (± 5.4)	57.8 (± 9.1)	47.8 (± 5.1)	60 (± 5.5)	59.2 (± 4.4)	44.2 (± 7)
CD40	29 (± 2.3)	28.7 (± 2.3)	25.6 (± 4.2)	26.4 (± 2)	25.6 (± 4.5)	28 (± 0.8)
CD83	18.4 (± 1.1)	20.7 (± 5.6)	18.2 (± 1.8)	20.5 (± 6.3)	18.4 (± 1.1)	21 (± 6.4)
TLR2	16.2 (± 1.8)	16.2 (± 1.6)	19.2 (± 3)	18.4 (± 3.6)	21.4 (± 6)	17 (± 1.2)
TLR4	29.3 (± 2.3)	28.4 (± 2.3)	33.7 (± 7)	29.2 (± 4.8)	31.8 (± 5.7)	33.8 (± 6.7)
PD-L1	25 (± 4.5)	23 (± 3.4)	22.6 (± 2.1)	23.4 (± 2.9)	29.4 (± 4.7)	29 (± 3.6)
PD-L2	17.4 (± 4.6)	17.4 (± 3.4)	17.4 (± 4.9)	18.6 (± 5.2)	21.8 (± 4.6)	18.8 (± 5.1)
DEC-205	42.8 (± 15.6)	42.8 (± 11.2)	41.2 (± 9.8)	46.4 (± 10.8)	53 (± 18.7)	36 (± 2.4)
CD64	29.8 (± 7)	29.9 (± 5.7)	24.7 (± 6.7)	22.6 (± 2.6)	22.8 (± 6.4)	23.3 (± 3.4)

**FIGURE 3:**
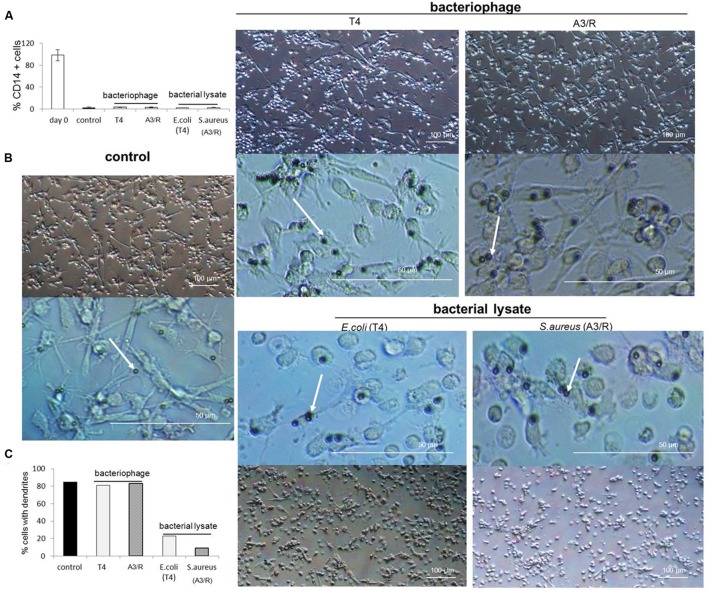
**Morphology of myeloid dendritic cells differentiated in the presence of bacteriophages and bacteriophage-generated bacterial lysates.** Human mDCs were differentiated from CD14^+^ monocytes during five-day cultures with IL-4 (100 ng/ml) and GM-CSF (250 ng/ml). The investigated preparations (T4 or A3/R bacteriophage, or the corresponding bacterial lysate) were added to wells on Day 0 of culture. In control cultures cells were treated with equal volumes of PBS. In all culture variants cells have lost the expression of CD14 during differentiation **(A)**. Morphology of mDCs differentiated in the presence of individual preparations is shown at lower and higher resolution. White arrows indicate microbeads attached to cell surface **(B)**. The presence of bacterial lysate of both T4 and A3/R phage resulted in a reduction in the number of dendrites in differentiated mDCs compared with mDCs from control cultures and mDCs generated in the presence of purified phage preparations **(B,C)**.

Moreover, we did not observe any visible changes in morphology of mDCs generated in cultures to which phages were added compared with control cultures; in particular, we found no reduction in the number of cells with long dendrites, a hallmark of typical DC morphology (**Figure 3**).

**FIGURE 4:**
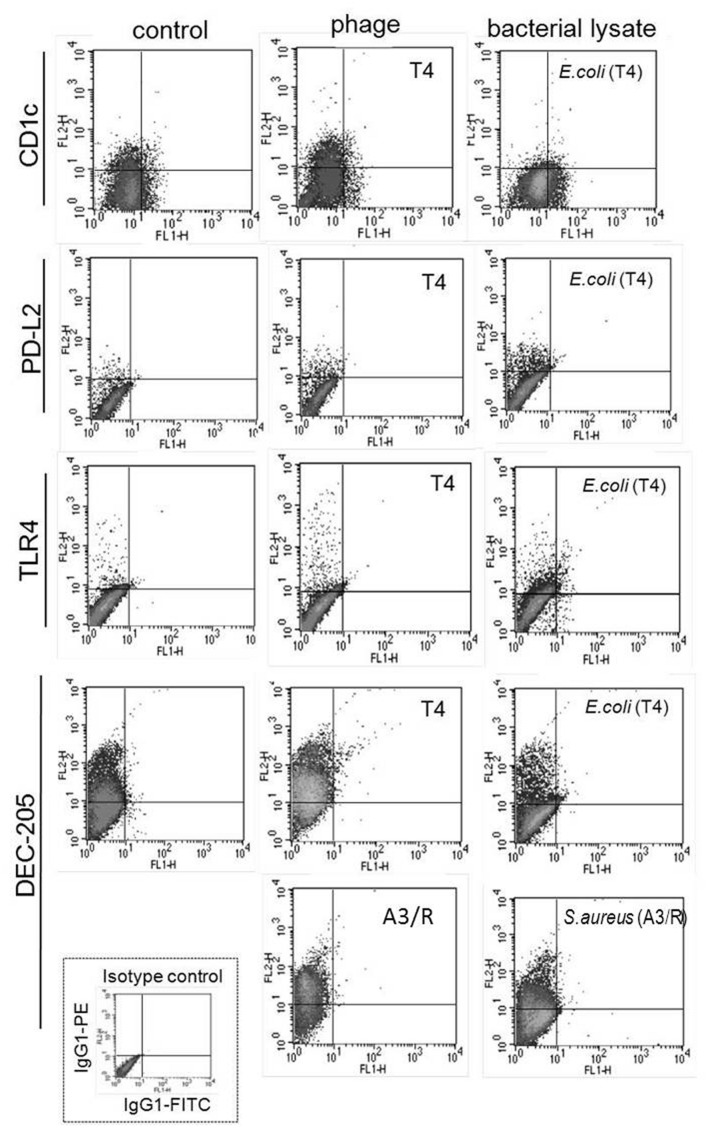
**Representative results of analysis of the expression of surface markers of mDCs differentiated in the presence of bacteriophage-generated bacterial lysates.** Shown are representative density plots for markers for which statistically significant differences were found between mDCs differentiated in the presence of bacterial lysates and mDCs from control cultures (treated with PBS). In addition, the figure shows representative density plots for mDCs differentiated in the presence of bacteriophages. Anti-CD1c, anti-PD-L2, anti-TLR4, and anti-DEC-205 monoclonal antibodies were conjugated with phycoerythrine (PE). Shown is also a representative density plot for isotype control (IgG-PE).

Unlike purified bacteriophage preparations, phage-generated bacterial lysates affected the expression of some of the investigated markers. First, the percentages of DEC-205- and CD1c-positive cells were significantly reduced in mDCs differentiated in the presence of lysate of *E. coli* compared with mDCs from control cultures (*p* = 0.001 and *p* = 0.002, respectively; **Figures 1**, **2**, and **4**). Moreover, the percentages of TLR4- and PD-L2-positive mDCs were significantly higher in cultures to which the lysate was added compared with control cultures (*p* = 0.012 and *p* = 0.018, respectively; **Figures 1** and **4**); however, no significant difference was observed in the percentages of TLR4- and PD-L2-positive cells between mDCs generated in the presence of the lysate compared with mDCs differentiated in the presence of peptone water (data not shown).

We also found that in mDCs differentiated in the presence of lysate of *S. aureus* the percentage of DEC-205-positive cells was significantly reduced compared with mDCs from control cultures (*p* = 0.016; **Figures 2** and **4**). However, neither of the investigated lysates significantly affected the mean channel fluorescence values for individual markers (**Table 1**).

Representative density plots for markers for which statistically significant differences were found between mDC differentiated in the presence of lysates and mDCs from control cultures are shown in **Figure 4**.

We also observed that both lysates significantly reduced the number of cells with typical DC morphology (long dendrites) compared to control cultures (**Figure 3**).

Apart from the expression of main DC markers, we also investigated the production by mDCs of IL-12 p70. However, the level of IL-12 was undetectable in culture supernatants of mDCs generated in the presence of both purified phage preparations and lysates of *E. coli* and *S. aureus*, as well as in control cultures (data not shown).

## Discussion

The overall objective of this study was to evaluate the effects of T4 and A3/R bacteriophages on the differentiation of human mDCs from monocytes. In *in vivo* settings phages may affect function of immune cells not only by direct interactions, but also indirectly, by different components of bacterial cells released from lysed bacteria. Therefore, our study included experiments to investigate both direct effects of bacteriophage particles on mDCs and the influence of phage-generated bacterial lysates. Such lysates are also used in the treatment of bacterial infections at some centers of phage therapy (Mi˛dzybrodzki et al., 2012).

As model phages we employed T4 and A3/R. T4 is a classic phage that has been extensively characterized at the genetic and molecular level (Leiman et al., 2003). It belongs to the T4-like viruses genus of the *Tevenvirinae* subfamily of *Myoviridae* (Adriaenssens et al., 2012). Its dsDNA genome is 168 kb long and contains 278 open reading frames (ORFs) (Miller et al., 2003; Petrov et al., 2010). A3/R is a novel bacteriophage that was described recently (Łobocka et al., 2012). It is a strictly lytic, polyvalent staphylococcal phage from the Twort-like genus of the *Spounavirinae* subfamily of myoviruses. The dsDNA genome of A3/R is 132 kb long and contains 196 ORFs (GenBank Acc. No. JX080301).

We found that both the percentages of cells expressing individual markers and the mean channel fluorescence values for these markers were similar in mDCs generated in the presence of phages and in control cultures. These results indicate that T4 and A3/R do not adversely affect the differentiation of mDCs and the expression of molecules essential for some of DCs’ main functions, especially a role in T cell activation and phagocytosis. The data obtained for T4 bacteriophage are in line with the results of Miernikiewicz et al. (2013) who showed that this phage does not significantly affect the expression of CD40, CD80, CD86, and MHC class II molecules on murine BMDCs *in vitro*. Likewise, in the study by Pajtasz-Piasecka et al. (2008), incubation with T4 did not substantially influence the expression of BMDC maturation markers including CD80, CD86, CD40, CD54, as well as class I and class II MHC. Potential effects on DCs of the other of the investigated bacteriophages – A3/R – have not been evaluated previously. In other studies, *Cronobacter sakazakii* ES2 phage was found to increase the expression of CD40, CD86, and MHC class II molecules on BMDCs (An et al., 2014), while filamentous bacteriophage fd did not affect the expression of CD40, CD80, CD86, MHC class I and MHC class II molecules on these cells (Sartorius et al., 2011). Thus individual phages can exert different effects on DCs.

It needs to be stressed that all previous studies on the interactions between bacteriophages and DCs have been conducted on BMDCs and their results cannot be simply extrapolated to human DCs. This results from the fact that there are substantial differences between murine and human DCs (Robbins et al., 2008; Dutertre et al., 2014; Schlitzer et al., 2015). To the best of our knowledge, our study is the first to evaluate the effects of phages on human DCs. In addition, a panel of DC markers analyzed in our study is considerably wider than the panels used in previous studies. For example, our study is the first to examine the effects of phages on the expression of some molecules associated with differentiation of DCs and their role in the activation of T cells (CD1c, CD11c, PD-L1, PD-L2, TLR2, TLR4, and CCR7), as well as of phagocytosis receptors (CD64 and DEC-205).

Recently, a specific subpopulation of DCs, called inflammatory DCs (infDCs) was described (Serbina et al., 2009; Segura and Amigorena, 2013; Dutertre et al., 2014; Schlitzer et al., 2015). InfDCs are generated from monocytes especially during inflammatory reactions (including those accompanying infections). The differentiation of monocytes into infDCs occurs at sites of ongoing inflammation. Thus, a finding that phages do not adversely affect the differentiation of monocytes into mDCs *in vitro* suggests that bacterial viruses should not inhibit the generation of infDCs *in vivo*; however, this notion needs to be verified by further studies.

It is known that immature DCs do not produce IL-12; the production of this cytokine is a feature of antigen-activated mature DCs (Albrecht et al., 2008; Turnquist et al., 2010). Thus, to evaluate whether bacteriophages can activate immature DCs, we determined the production of IL-12 by mDCs generated in the presence of T4 and A3/R. However, the level of IL-12 was undetectable in culture supernatants from mDCs generated in the presence of bacteriophages, as well as in control cultures. This confirms that phages do not activate immature mDCs.

To evaluate whether the products of phage-induced lysis of bacterial cells can affect DCs, we used unpurified *E. coli* and *S. aureus* lysates generated *in vitro* as a result of infection of bacteria by T4 and A3/R, respectively. Bacterial lysates appear to be suitable model preparations for studying the effects of products of phage-induced lysis of bacterial cells generated *in vivo*. Moreover, investigating the effects of lysates is of practical importance because such preparations are used at some centers of phage therapy in the treatment of bacterial infections (Mi˛dzybrodzki et al., 2012). We found that bacterial lysate of T4 phage significantly decreased the percentages of DEC-205- and CD1c-positive mDCs. The percentage of DEC-205-positive cells was also significantly reduced in mDCs generated in the presence of lysate of *S. aureus*, while the expression of all other markers was unaffected. Remarkably, neither of the studied lysates substantially affected the mean channel fluorescence value for any marker. Furthermore, neither of the lysates stimulated the production by mDCs of IL-12.

DEC-205 (CD205) is a type I C-type lectin-like receptor involved in antigen uptake, processing, and presentation. Antigens endocytosed via DEC-205 were shown to enter both MHC class I and MHC class II antigen presenting pathways and to be consequently presented to both CD4^+^ and CD8^+^ T cells. Recently DEC-205 was found to recognize ligands expressed during apoptosis and necrosis by different cell populations, which confirms a role of this receptor in the induction of both central and peripheral tolerance to self-antigens (Shrimpton et al., 2009). CD1c belongs to Group 1 CD molecules. Its main function is the presentation of lipid antigens to different subpopulations of T cells. Lipids presented by CD1c include sphingomyelin, phosphatidylcholine, phosphatidylinositol, sulfatides, and acylated lipopeptides. CD1c was found to be capable of presenting as antigens both endogenous lipids and some components of *Mycobacterium tuberculosis* (Adams, 2014). It should be noted that CD1c^+^ cells generated and investigated in our study are more related to monocyte-derived cells than to *bona fide* DCs (Guilliams et al., 2014).

Differences in the percentages of mDCs expressing CD1c and DEC-205, as well as in morphology of differentiating DCs suggest the presence in the lysate of T4 phage of factor(s) inhibiting the differentiation of DCs from monocytes. These factor(s) might inhibit DEC-205-mediated phagocytosis by DCs as well as presentation of lipid antigens by CD1c. However, whether the products of phage-induced lysis of bacterial cells can affect functions of DCs mediated by these molecules should be verified experimentally.

The presence of bacterial lysate of A3/R phage resulted in a decrease in the percentage of DCs expressing DEC-205 (and thus possessing a capacity for phagocytosis) and a lower number of cells with typical DC morphology.

Given that the described changes in the expression of DC markers were found only in cells cultured with phage-generated bacterial lysates, and did not occur in cells treated with purified bacteriophages, it is apparent that they were caused not by phage virions themselves, but rather by some components of bacterial cells present in the lysates. These results indicate that some products of phage-induced lysis of bacteria also could affect function of DCs *in vivo*. However, at this stage of our research we wanted to evaluate whether bacteriophage-generated bacterial lysates can have any effect on the differentiation of DCs. Further studies should be conducted to identify the component(s) of lysates that affect the expression of mDCs markers. Given that some significant changes in the expression of mDCs markers (as well as in morphology of mDCs) were induced also by *S. aureus* lysate, it is unlikely that LPS was the sole bacterial component responsible for these effects. Apart from LPS (present in *E. coli* lysate) also other bacterial components could influence the differentiation of DCs.

The interactions between the products of the phage-induced lysis of bacteria and immune cells might be important especially in the gut where phages are present in very large numbers and constitute the primary factor to regulate the growth of the microbiota (Letarov and Kulikov, 2009; Mills et al., 2013; Dalmasso et al., 2014). Lysis of bacterial cells can also occur in patients with bacterial infections treated with phage preparations. In fact, phage therapy is currently considered one of the most promising ways of treatment of antibiotic-resistant infections (Kutter et al., 2014; Wittebole et al., 2014).

The present study focused on the first stage of the development of DCs, that is the differentiation of immature DCs from monocytes. To extend knowledge on the interactions between phages and DCs, studies should be performed to evaluate the effects of phage preparations on further stages of DCs development, in particular their maturation and function of mature DCs, especially their role in the regulation of adaptive immune responses. Our results indicate that such studies should involve both purified phages and phage-generated bacterial lysates because these two types of preparations can have different effects on DCs.

## Author Contributions

KB performed the experiments, analyzed data, and contributed to drafting of the manuscript. JB analyzed data and contributed to drafting of the manuscript. MZ, MP and MM performed the experiments. BW-D analyzed data. GK-K conceived the study, analyzed data and contributed to drafting of the manuscript. AG conceived the study and contributed to drafting of the manuscript. All authors approved the final version of the manuscript.

## Conflict of Interest Statement

AG and BW-D are co-inventors on patents covering preparation of therapeutic phages owned by the IIET, Wrocław, Poland. The institutions that funded the study had no role in the design of the study; in the collection, analyses, or interpretation of data; in the writing of the manuscript, and in the decision to publish the results. Neither the authors nor their institutions received payment or services from a third party for any aspect of the submitted work. All the other authors declare that the research was conducted in the absence of any commercial or financial relationships that could be construed as a potential conflict of interest.
